# Syringe pump extruder and curing system for 3D printing of photopolymers

**DOI:** 10.1016/j.ohx.2021.e00175

**Published:** 2021-02-03

**Authors:** Cory Darling, Damon A. Smith

**Affiliations:** a Department of Mechanical Engineering, University of New Orleans, United States; b Advanced Materials Research Institute (AMRI), University of New Orleans, United States

**Keywords:** 3D printing, Additive manufacturing, Bioprinter, Photopolymer, UV resin

## Abstract

Development of new additive manufacturing materials often requires the production of several batches of relatively large volumes in order to print and test objects. This can be difficult for many materials that are expensive or difficult to produce in large volumes on the laboratory scale. Bioprinter systems are advantageous in this regard, however, commercial systems are expensive or do not have the ability to use photopolymers. Herein, we outline a Syringe Pump Extruder and Curing System (SPECS) modification for inexpensive filament-based 3D printers which enables the use of standard bioplotter materials and photopolymers. The system is capable of using multiple syringe volumes and needle sizes that can be quickly and easily exchanged. The SPECS modification is demonstrated using a Prusa i3 mk3 fused filament fabrication printer to print several 3D objects and films using stereolithography (SLA) photopolymer resin. Geometric accuracy in the X, Y, and Z directions was ±0.1 mm using a 5 ml syringe, 22-gauge needle, and commercial SLA resin. The SPECS system could be of great benefit for laboratories pursing material development in the area of additive manufacturing.

## Graphical abstract

**Figure:**
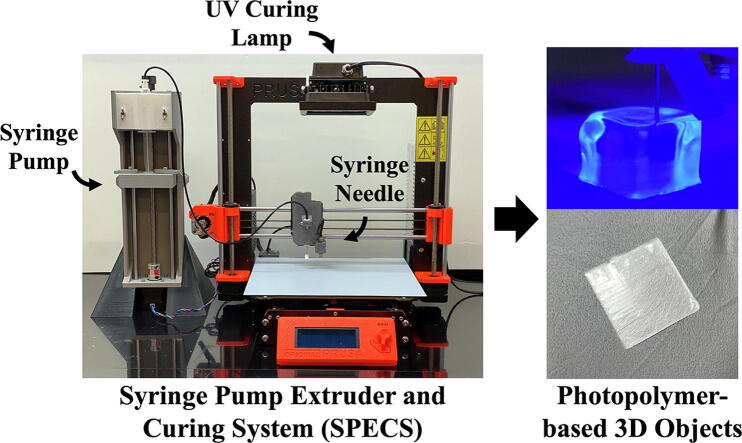

**Specifications table**

**Table:** 

Hardware name	Syringe Pump Extruder and Curing System (SPECS)
Subject area	Educational Tools and Open Source Alternatives to Existing Infrastructure
Hardware type	3D Printer Modification
Open Source License	CC-BY-SA 4.0
Cost of Hardware	$277.24
Source File Repository	https://doi.org/10.17632/cv9cfj39b7.1

## 1 Hardware in context

### 1.1 Introduction

**Fig. 1:**
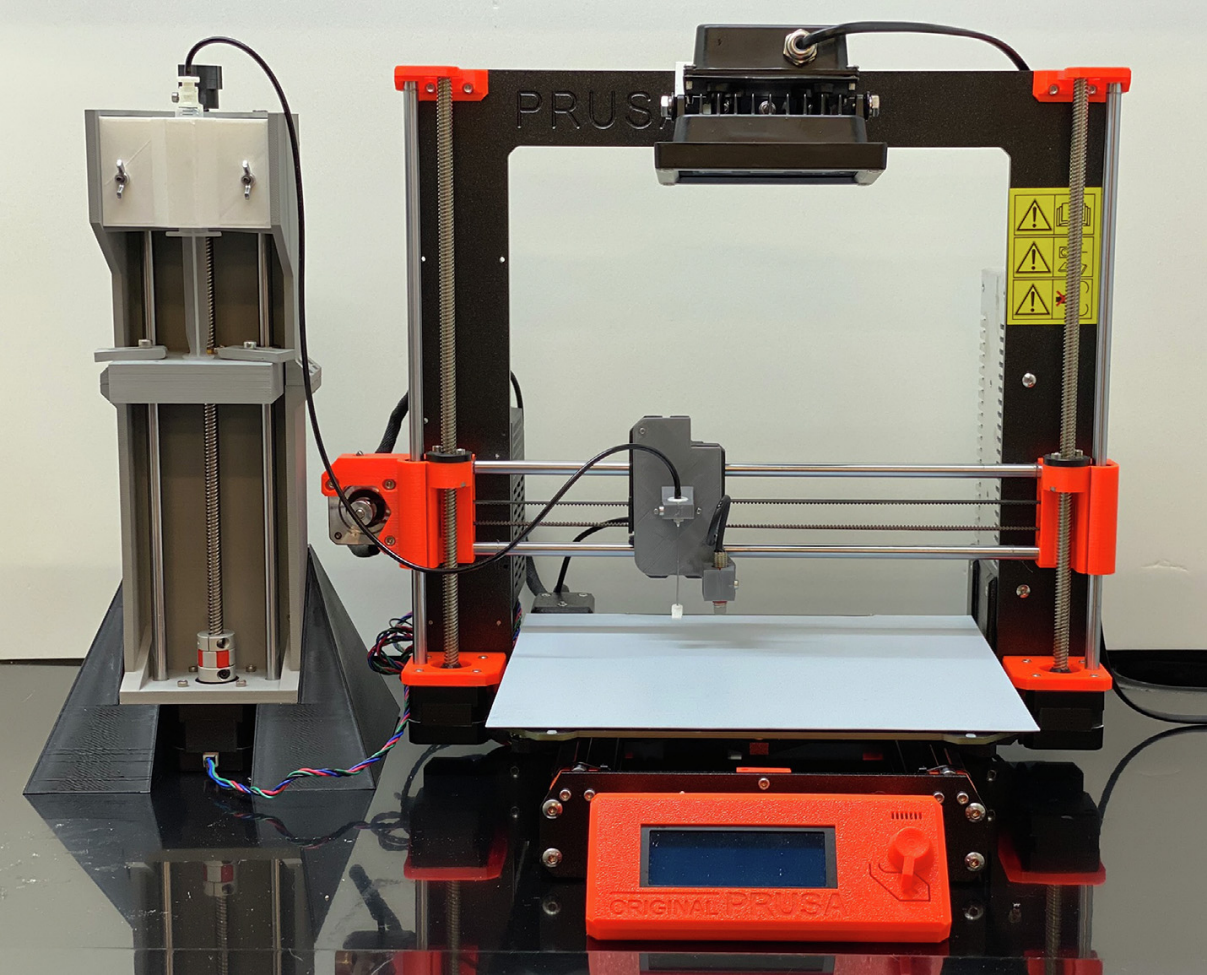
Prusa i3 mk3 desktop FFF 3D printer modified for UV resin build materials using the described Syringe Pump and Curing System (SPECS) hardware. The system can hold a variety of different syringe volumes and needle sizes for extrusion of build materials on a Teflon print bed.

Research and development of new materials that are compatible with additive manufacturing (AM) processes have received a dramatic increase over the last decade [1], [2], [3]. While AM systems are advantageous with respect to the rapid production of objects and parts with unprecedented structural complexity, the range of materials currently available is more limited than many traditional manufacturing methods [3]. Therefore, there is significant motivation to improve and expand the available pallet of materials and properties that are available to designers and engineers. Much of the research in AM material development has focused on composites and various polymer blends [1], [2], [3]. Materials research for fused filament fabrication (FFF) systems have received the majority of the research focus [4]. FFF systems are inexpensive but require the use of additional extrusion equipment to produce filament when studying new materials. Filament extruders, such as the Filabot EX2, can be purchased for less than $3000 USD. However, these extrusion systems typically produce a meter or greater of inhomogeneous filament at the start of the extrusion process when compounding materials. This makes the exploration of some materials difficult, particularly when high concentrations are needed [5]. For example, harnessing the novel and size-tunable properties of nanoparticle materials for FFF processes has shown significant promise for expanding the applicability of AM in biomedicine, electronics, aerospace and many other fields [1], [2], [4], [5], [6]. However, producing the necessary quantities of these materials for FFF processes can be challenging on a laboratory scale and waste needs to be minimized. Systems that mix materials prior to extrusion can significantly reduce this waste, but this equipment can cost greater than $100,000 USD. Stereolithography (SLA) systems are now approaching the cost of FFF systems, but SLA printers also require a large material volume that can make material development even less feasible than FFF [7]. For example, the Formlabs Form 2 SLA system requires a tray filled with 200 ml of photopolymer resin. Producing a series of formulations of this volume would be a challenge for many materials. Therefore, the primary motivation for the described hardware development is to meet the need for a low-cost and flexible AM system that can be used to explore a large range of build materials, including photopolymers, at milliliter volumes in order to facilitate research and development. This is accomplished by modification of an inexpensive commercially available FFF system with a Syringe Pump Extruder and Curing System (SPECS) for use with photopolymers and general liquid and gel feedstock (Fig. 1). Several approaches have been documented for the production of general use open-source syringe pumps [8], [9], [10], [11], [12], [13], [14]. There are also some open-source syringe pumps that have been developed for the production of low-cost syringe-based AM systems [15], [16], [17], [18]. However, these systems do not incorporate UV curing for the use of small volumes of photopolymers.

### 1.2 Material compatibility and volume

While the system validation described herein focuses on the use of UV photopolymer resins, the SPECS modification can also be used for liquids, pastes, and gels that are commonly employed in pump-based AM systems such as bioprinters and food and paste printers. Recently, Kretzschmar et al. documented an AM system that also incorporated UV curing along with a paste extrusion process [18]. The system described herein has similar capabilities but is implemented as a low-cost FFF system modification that does not require any expensive fabrication tools or software. To the best of our knowledge, no other open-source AM systems for extrusion of photopolymers currently exist in the literature.

There are numerous research areas that can benefit from a 3D printer with variable volume capabilities. The SPECS modification meets the demand for an AM system for testing small batches of photopolymer build material by using a detached syringe pump which can hold a wide range of syringe volumes. The hardware provided includes 3D printed holders for 5 ml and 60 ml syringe volumes. This makes it possible to initially test small volumes of material and scale up to the printing of larger objects, if needed.

### 1.3 Cost

Extrusion-based AM systems that can use liquids, gels, resins, or pastes as feedstock are ideal for researching advanced materials in small batches [3]. However, commercially-available systems are typically expensive bioprinters that can cost greater than $100,000 USD. The current leading company distributing commercial grade bioprinters is EnvisionTEC, with manufacturer grade machines ranging from $100,000 to $250,000 USD. These systems use a similar gantry system and build stage mechanism found in FFF systems, but deliver feedstock using a pneumatic or syringe pump. Therefore, conversion of the filament extruder to a pump delivery system on an inexpensive FFF printer is a promising alternative to commercial bioprinters [16]. Herein, the SPECS modification of the Prusa i3 Mk3 FFF printer has a cost of $277.24 USD. Though the modification is based on the use of a Prusa printer, the detached syringe pump design employed can be easily adapted for use with most consumer-grade FFF printers.

### 1.4 Performance

The SPECS modification of the Prusa i3 Mk3 preserves its bed leveling function and repurposes the filament drive motor to operate a syringe pump. Similar to the open-source system developed by Pusch et al., this allows for control of the flow rate and the application of a consistent extrusion pressure with the ability to reverse flow direction [16]. Due to the direct drive nature of the syringe pump, this system is capable of extrusion speeds and accuracies similar to modern FFF 3D printers, including features such as retraction to allow for a clean deposition of fluid material without over extrusion, dripping, oozing, or smearing of the fluid. The ability to use a standard slicing software (Prusa Slicer) allows the user to fine tune almost every aspect of the print speed, accelerations, fluid path width, and infill pattern. The simplistic nature of the conversion gives the added benefit of easily swappable syringes and needles with very few parts and hardware. The Bowden configuration of the extruder, which uses a detached pump connected to the needle by plastic tubing, adds no addition mass to the gantry system and, therefore, retains the original precision and dimensional capabilities of the printer. The retention of the automatic bed leveling function allows for high first layer Z-height accuracy and the ability to print thin films only constrained by the minimum step height of 0.01 mm. An inexpensive UV curing lamp is attached to the printer frame using a 3D printed clamp in order to cure the UV photopolymer resin during the printing process. This position illuminates the entire build plate with UV light. Curing time and resolution are determined by the specifications of the photopolymers used. Experiments with Formlabs SLA Clear Resin at a print speed of 20 mm s^−1^ and a layer height of 0.2 mm showed that curing was sufficient to build 3D objects.

## 2 Hardware description

General description of hardware. The SPECS modification consists of three parts: (1) the syringe pump, which delivers the fluid build material, (2) the extrusion head that holds the syringe tip and the bed leveling sensor, and (3) the UV lamp assembly, which is used to attach the UV lamp to the printer frame and is required to cure photopolymers during the build process.

### 2.1 Syringe pump

The syringe pump design is based on standard laboratory syringe pump hardware in which multiple syringe sizes can be used. The current maximum size syringe that will fit into the syringe pump with its entire volume being utilized is 60 ml and the minimum syringe size is 5 ml. This allows for either high volume prints or small prints where conservation of materials is important, material batches are small, volatile materials must be used quickly and in small volumes, or material availability is low.

The syringe pump is also designed for simplicity. All 3D printed parts can be printed by the Prusa i3 mk3 prior to the conversion, can be printed with no supports, and the entire pump only consists of eight 3D printed parts and minimal addition hardware. The syringe pump utilizes the existing extruder stepper motor and alterations to the slicing software allow for a fine level of adjustment for fluid flow rates.

### 2.2 Extrusion head

The extrusion head is a simple design which preserves the original position of the automatic bed leveling sensor and extrusion location. The clamp-style design of the needle retention location makes it easy to adjust the needle height and change needle sizes as needed. Due to the high-quality construction of the Prusa i3 mk3, little to no vibration is present in the needle, even at high print velocities. The extrusion head utilizes the existing mounting holes for the stock extrusion head as well as cable routing location for the automatic bed leveling sensor.

### 2.3 UV lamp assembly

The UV lamp assembly allows for the lamp to be mounted on the top cross member of the Prusa i3 mk3 without interfering with the printer’s function.

### 2.4 Key aspects of the hardware

The SPECS modification is of interest to researchers developing new materials for fluid-based AM processes. Additionally, the printer allows for the unique ability to use photopolymers.

• The FFF printer modification employs a detached syringe pump which can be used to adapt most consumer-grade printers.
• The modification preserves the bed leveling functionality of the Prusa i3 Mk3 printer allowing for accurate thin film printing and high resolution.
• The existing stepper motor is used to drive the syringe pump.
• The slicer software, Prusa Slicer, is open source and can be easily altered to be fully integrated with this fluid deposition system.
• Syringes with different volumes and syringe tip sizes can be quickly swapped out allowing for the use wide range of materials with varying viscosities and particle additive inclusion sizes.
• A multitude of needle sizes can be used for deposition.
• All primary parts of the conversion can be printed with the Prusa i3 mk3 prior to the conversion.

## Design files

Engineering drawings, renderings, and exploded diagrams of the conversion hardware are included in the table below.

Design Files Summary

**Table:** 

Design file name	File type	Open source license	Location of the file
Full Assembly	CAD & STL	CC-BY-SA 4.0	https://doi.org/10.17632/cv9cfj39b7.1
Pump Frame – Top	STL	CC-BY-SA 4.0	https://doi.org/10.17632/cv9cfj39b7.1
Lead Screw Knob	STL	CC-BY-SA 4.0	https://doi.org/10.17632/cv9cfj39b7.1
Large Syringe Clamp	STL	CC-BY-SA 4.0	https://doi.org/10.17632/cv9cfj39b7.1
Small Syringe Clamp	STL	CC-BY-SA 4.0	https://doi.org/10.17632/cv9cfj39b7.1
Plunger Plate	STL	CC-BY-SA 4.0	https://doi.org/10.17632/cv9cfj39b7.1
Pump Frame-Bottom	STL	CC-BY-SA 4.0	https://doi.org/10.17632/cv9cfj39b7.1
Plunger Clamps	STL	CC-BY-SA 4.0	https://doi.org/10.17632/cv9cfj39b7.1
Extruder Head	STL	CC-BY-SA 4.0	https://doi.org/10.17632/cv9cfj39b7.1
Needle Tip Shield	STL	CC-BY-SA 4.0	https://doi.org/10.17632/cv9cfj39b7.1
UV Light Mount	STL	CC-BY-SA 4.0	https://doi.org/10.17632/cv9cfj39b7.1
Syringe Pump Stand	STL	CC-BY-SA 4.0	https://doi.org/10.17632/cv9cfj39b7.1

### 3.1 Brief description of each part

**Full Assembly:** An STL and CAD file of the complete assembly is provided that includes all 3D printable parts.

**Fig. 2:**
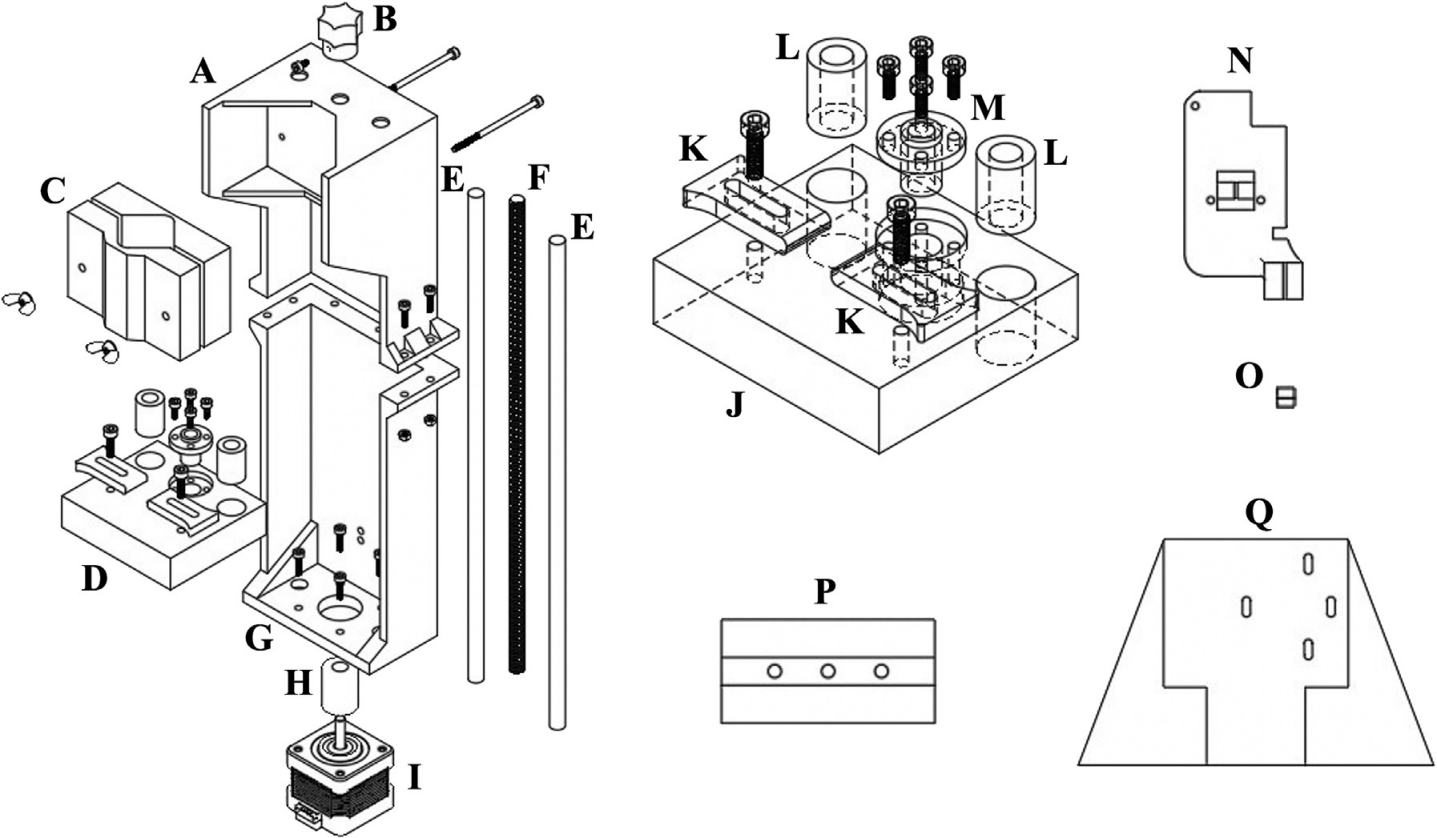
Exploded diagram of the major components of the assembly showing the (A) pump frame (top), (B) lead screw knob, (C) syringe clamp, (D) plunger plate, (E) guide rails, (F) lead screw, (G) pump frame (bottom), (H) lead screw coupler, (I) drive motor, (J) plunger plate (close-up view), (K) plunger clamps, (L) linear bearings, (M) lead screw nut, (N) extruder head, (O) syringe tip shield, (P) UV light mount, and (Q) syringe pump stand.

**Pump Frame – Top (**Fig. 2A**):** The top section of the syringe pump frame.

**Lead Screw Knob (**Fig. 2B**):** The lead screw knob attaches to the top of the driving lead screw and allows for easy manual manipulation of the syringe pump.

**Large and Small Syringe Clamp (**Fig. 2C**):** Clamp inserts are designed to securely hold both a 60 ml and a 5 ml standard syringe. Additional inserts for different syringe volumes may easily be printed by the user.

**Plunger Plate (**Fig. 2D**):** The plunder plate is the primary translating component of the syringe pump. The plunger of the syringe is clamped to this plate with the plunger clamps and the translation of this plate extrudes or retracts the build material.

**Pump Frame – Bottom (**Fig. 2G**):** The bottom section of the syringe pump frame. The bottom of the pump frame also has holes to optionally mount the syringe pump directly to the Prusa i3 mk3 on the back side of the machine above the control board housing.

**Plunger Clamps (**Fig. 2K**):** Small lever clamps that secure the syringe plunger to the plunger plate and allow for retraction of the syringe.

**Extruder Head (**Fig. 2N**):** The extruder head replaces the existing extrusion head of the Prusa i3 mk3. The needle and the automatic bed leveling sensor are mounted on this component.

**Syringe Tip Shield (**Fig. 2O**):** The syringe tip shield prevents clogging or curing on the tip of the needle when exposed to the UV curing lamp during printing.

**UV Light Mount (**Fig. 2P**):** Mounting bracket to attach the 6 W 405 nm UV lamp to the Prusa i3 mk3 top cross-member.

**Syringe Pump Stand (**Fig. 2Q**):** Optional 3D printed part that allows the syringe pump to be mounted separately from the Prusa i3 mk3.

## 4 Bill of materials

**Table:** 

Designator	Component	Number	Cost per unit – USD	Total cost – USD	Source of materials	Material type
8 mm Lead Screw and Nut	OS16206	1	$10.60	$10.60	Amazon	Stainless Steel
LM8UU Linear Bearings	a13081400ux0267	2	$18.12	$18.12	Amazon	Carbon Steel
8 mm Linear Motion Guide Rails	DIA8SF80400	2	$31.60	$31.60	Amazon	Chromed Steel
Flexible Shaft Coupling – 5–8 mm	BE-032-fba	1	2/pk $13.19	$13.19	Amazon	Aluminum Alloy
M3 × 12 mm Hex Bolts	91290A117	11	100/pk 0.09	8.67	McMaster Carr	Alloy Steel
M3 Hex Nuts	90592A085	11	100/pk 0.009	0.88	McMaster Carr	Steel
M4 × 12 mm Hex Bolts	91290A148	3	100/pk 0.09	9.22	McMaster Carr	Alloy Steel
M3 × 8 mm Hex Bolts	91290A113	5	100/pk 0.07	7.37	McMaster Carr	Alloy Steel
M3 × 60 mm Hex Bolts	91290A181	2	25/pk 0.34	8.57	McMaster Carr	Alloy Steel
M3 Wing Nuts	94545A210	2	25/pk 0.49	12.16	McMaster Carr	Steel
M3 Tapered Heat-set Threaded Inserts	94180A353	2	50/pk 0.19	9.37	McMaster Carr	Brass
6 W 405 nm UV Resin Curing Lamp	B07ZQ3TYR2	1	$16.99	$16.99	Amazon	–
UV Resistant Tubing, 1/16″ ID, 1/8″ OD	R-3400	2 + feet	50′ $25.67	$25.67	Amazon	Tygon
Female Luer Lock to 1/16″ Hose Barb Connector	AO-45502-00	1	25/pk $17.15	$17.15	Amazon	Nylon
Male Luer Lock to 1/16″ Hose Barb Connector	AO-45505-00	1	25/pk $11.90	$11.90	Amazon	Nylon
Teflon Sheet with Adhesive Backing, 1/32″ thick, 12 × 12″	TEFLON12x12-3M	1	$39.95	$39.95	Amazon	Teflon
2″, 22 Gauge, Flat Tip Syringe Needle	14-815-610	1	6/pk $35.83	$35.83	Fisher Scientific	Stainless Steel
24″ × 12″, 1/8″ Thick Laser Shielding (Optional)	Shielding-445-24	2	$33.99	$67.98	J Tech Photonics	Acrylic
24″ × 24″, 1/8″ Thick Laser Shielding (Optional)	Shielding-445-24-24	1	$79.99	$79.99	J Tech Photonics	Acrylic
Folding Butt Hinges, 2-2/5″ × 1-1/2″ (Optional)	QX-AZ18061307	1	6/pk $7.99	$7.99	Amazon	Metal

## 5 Build instructions

WARNING: UV EYE PROTECTION AND UV SHIELDING SHOULD BE USED TO PROTECT USERS FROM UV EXPOSURE.

Begin by 3D printing all parts included in the Mendeley repository. It is recommended that these parts are printed using PLA with an infill above 60% and a wall thickness above three to reduce the likelihood of part failure or deformation. These parts can be printed on the Prusa i3 mk3 prior to its disassembly and can be printed with no support material. The Mendeley repository includes some optional parts that can be printed if needed. These parts include:

• **Syringe clamps:** Two different types of syringe clamp are included for applications requiring up to 60 ml of material in a 60 ml syringe or a 5 ml syringes size for small material applications.
• **Syringe pump stand:** Used to mount the syringe pump separately from the Prusa i3 MK3. If the **syringe pump stand** is not printed, the syringe pump has mounting holes for mounting on the Prusa i3 MK3 frame.
• **UV lamp mounting bracket**: Used to attach a UV lamp to the Prusa i3 MK3 frame for UV photopolymer resin curing.

Tools required for assembly include:

• 3 mm Allen wrench
• 4 mm Allen wrench
• 5 mm box end wrench or socket
• Handheld drill or drill press
• 4 mm drill bit
• Vice or press
• Hack saw

### 5.1 Disassembly of the Prusa i3 MK3

In order to install the new extrusion head, the stock extrusion head on the Prusa i3 mk3 must be removed and some rerouting and removal of wires is required. The procedure is outlined below. For further diagrams and instructions on the disassembly of the Prusa i3 mk3, refer to the Mendeley repository for the which contains step-by-step images of the disassembly of the stock Prusa i3 mk3 and assembly of the conversion. Referencing the assembly manual for the Prusa i3 mk3 may also be useful in the disassembly process. The disassembly process is outlined below:

1. Ensure that the printer is unplugged.
2. Disassemble the print head until only the bearing retention plate that capture the linear bearings remain (Fig. 3A). Additional high-resolution pictures of the disassembly can be found in the Mendeley repository.

**Fig. 3:**
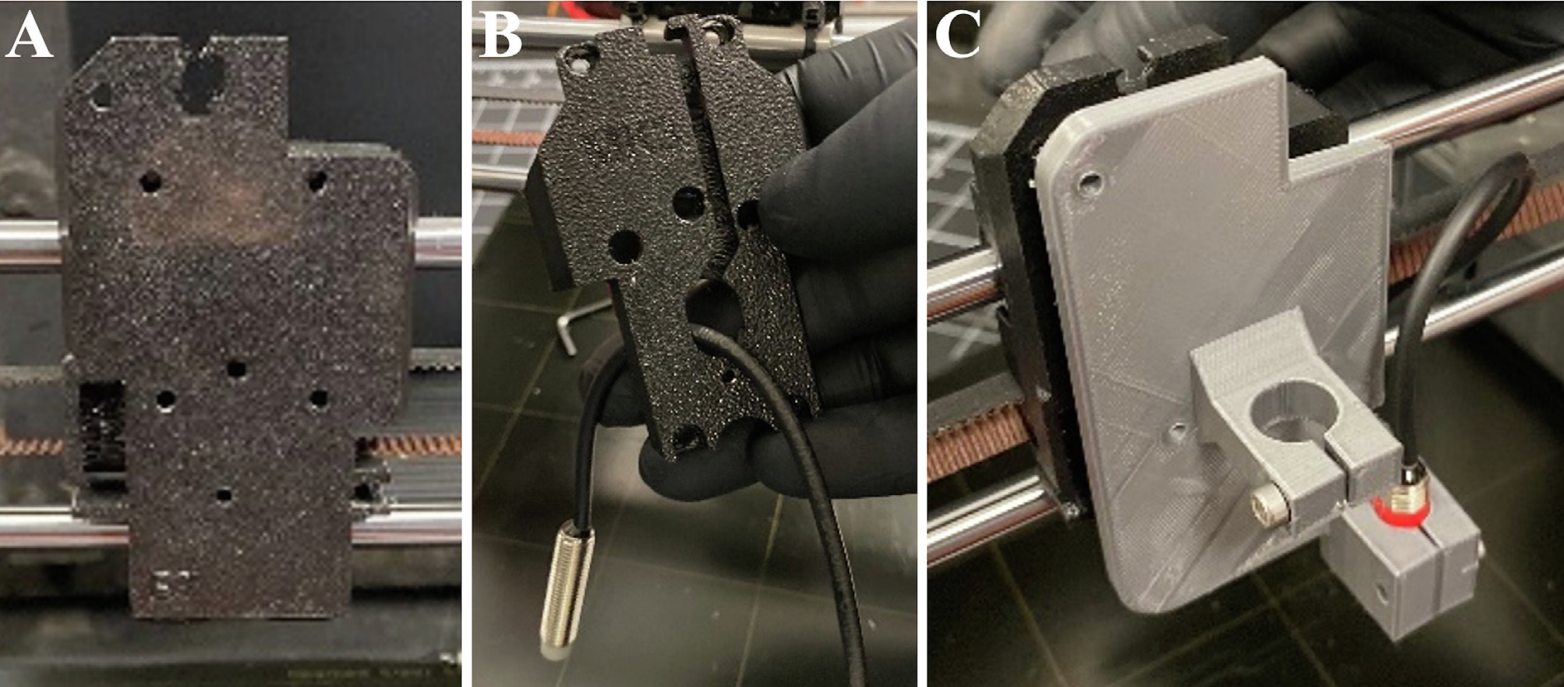
Assembly of the SPECS extrusion head. (A) Image of the Prusa i3 mk3 bearing retention plate after disassembly of the extrusion head. (B) The automatic bed leveling sensor fed through the hole in the backside of the linear bearing retention plate. (C) The 3D printed SPECS extrusion head bolted to the bearing retention plate.
3. Open the control box to expose the motherboard.
4. Disconnect all wires that connect to the print head from the motherboard. These include the:
  a. Part cooling fan
  b. Hot end fan
  c. Hot end wires
  d. Auto bed leveling sensor
  e. NEMA 17 extruder stepper motor
5. Clear all removed cables from the motherboard case
6. Thermistor bypass:
  a Due to the Prusa Mk3 i3 safety features, the hot end thermistor must remain plugged into the motherboard to allow operation.
  b To remove the hot end thermistor, loosen the grub screw next to the nozzle and remove the thermistor (white wires).
  c The thermistor can be covered with a piece of electrical tape and the thermistor wires can be coiled and tucked into the motherboard enclosure or another safe location.
  d The hot end assembly can be stored as it is not needed for this conversion.
7. Reconnect the extruder stepper motor and route cables out the bottom of the control box.
8. Tidy up cables and close control box.

### 5.2 Assembly of the extrusion head

1. You can now install the extrusion head onto the Prusa Mk3 i3. Locate the bed leveling sensor that was left connected to the motherboard and the disassembled extruder mount.
2. On the back of the extruder mount, remove the rear plate to expose the linear bearings.
3. Thread the bed leveling sensor through the cutout in the rear plate (Fig. 3B).
4. Re-attach the rear plate.
5. Secure the extruder head to the front of the extruder mount using 3 M3x8mm bolts (Fig. 3C).

### 5.3 Assembly of the syringe pump

1. Begin assembly by bolting the pump Frame – Top and pump Frame – Bottom together using 7 M3 × 12 mm bolts and M3 nuts (Fig. 4A).

**Fig. 4:**
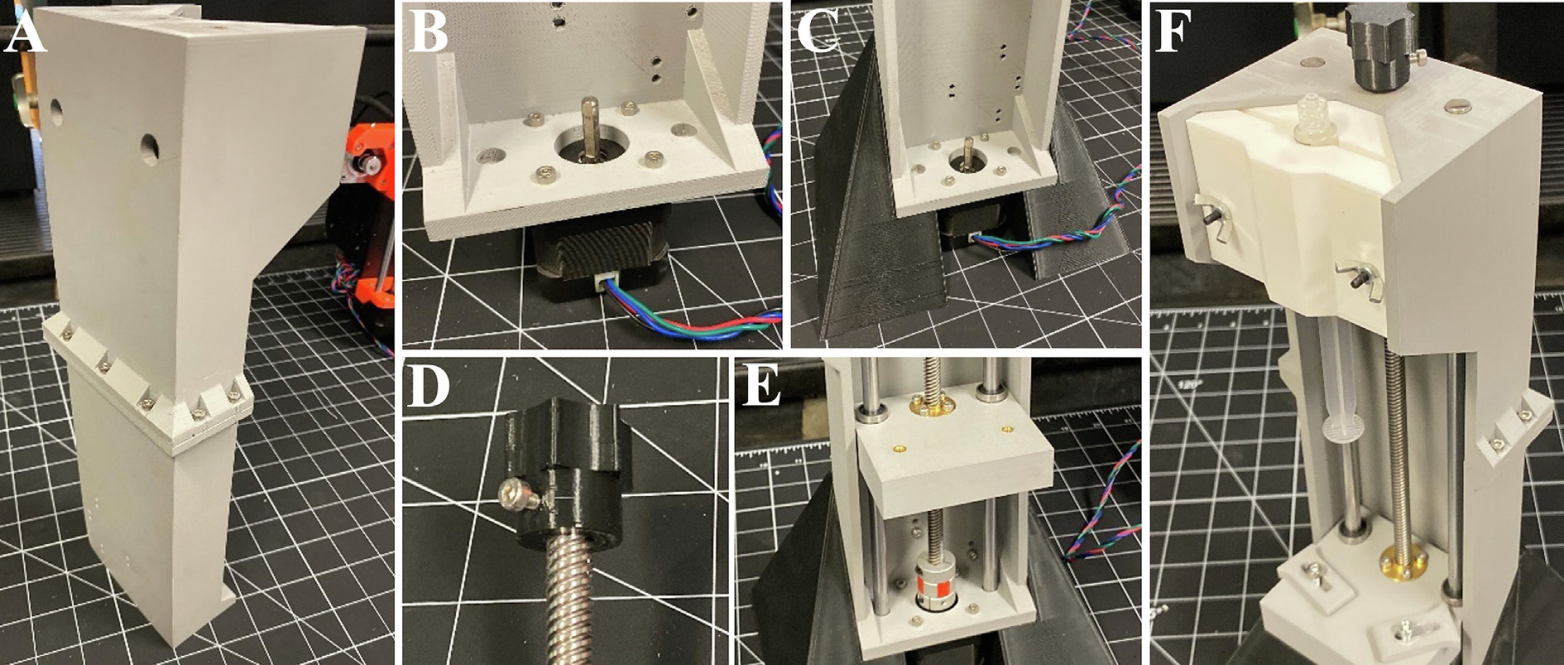
Assembly of the SPECS syringe pump. (A) Image of the assembled pump frame – top and pump frame – bottom. (B) Installation of the stepper motor into the base of the pump frame – bottom. (C) The syringe pump frame mounted onto the syringe pump stand. (D) Lead screw knob attached to the end of the lead screw. (E) The lead screw and linear rails threaded through the assembled plunger plate. The lead screw is also shown attached to the stepper motor using the shaft coupling. (F) Clamp insert for a 5 ml syringe by attached using 2 M3 wing nuts.
2. Use 4 M3 × 12 mm bolts to attach the NEMA 17 stepper motor to the bottom of the pump frame (Fig. 4B).
3. If the syringe pump stand is to be used, attached the pump frame to the syringe pump stand using 4 M3 × 12 bolts and 4 M3 nuts (Fig. 4C).
4. Insert the 2 M3 × 60 mm bolts through the access holes on the back of the pump frame and thread the bolts through the plastic and gently seat them (do not use washers on these bolts, it will interfere with the linear rails).
5. Insert the two M4 threaded inserts into the two 4 mm diameter holes in the plunger plate by heating the threaded insert using a heat gun, torch, butane lighter, or soldering iron and pressing them into the plastic until the insert is flush with the top of the plunger plate. Due to print settings, the holes for the threaded inserts may be slightly undersized. Use a 4 mm drill bit to clearance the holes if necessary.
6. Press the two linear bearings into the large diameter holes in the plunger plate (Fig. 2L) until they are centered. This can be done using a vise or press if the fit is tight.
7. Use four M3 × 8 mm bolts to attach the lead screw nut into the recess in the plunger plate. (Do not over tighten these bolts).
8. Install the lead screw coupler onto the NEMA 17 motor shaft and tighten the grub screw to secure the coupler to the motor.
9. Install the lead screw knob onto the top of the lead screw and secure the knob using an M3 × 8 mm bolt (Fig. 4D).
10. Insert the lead screw through the access hole in the pump frame- top and thread the lead screw through the lead screw nut in the plunger plate approximately halfway (Fig. 4E) to allow access to the coupler.
11. Lower the lead screw with the plunger plate attached down into the coupler and tighten the grub screw on the coupler to secure the lead screw.
12. Ensure that the plunger plate can move freely through the entire range of the lead screw by rotating the lead screw.
13. Insert the two 310 mm linear rails through the top of the pump frame so that they pass through the linear bearings and seat in the recesses on the bottom of the frame (Fig. 4E). NOTE: Unless linear rails are purchased at the correct length of 310 mm, the rails will need to be cut to length.
14. Again, check that the plunger plate can move freely through the entire range of the lead screw.
15. Use two M4 × 14 mm bolts to secure the plunger clamps to the plunger plate.
16. Optional: If desired, mount syringe to the backside of the Prusa Mk3 i3 support frame, above the motherboard enclosure using 4 M3 × 12 mm bolts and nuts.
17. Insert the desired syringe clamps and syringe into the upper slot in the syringe pump frame and tighten with 2 M3 wing nuts (Fig. 4F).

### 5.4 Syringe needle, UV lamp, and Teflon film installation

1. Attach the UV resistant tubing to the syringe using a female Luer lock hose barb connector (Fig. 5A).

**Fig. 5:**
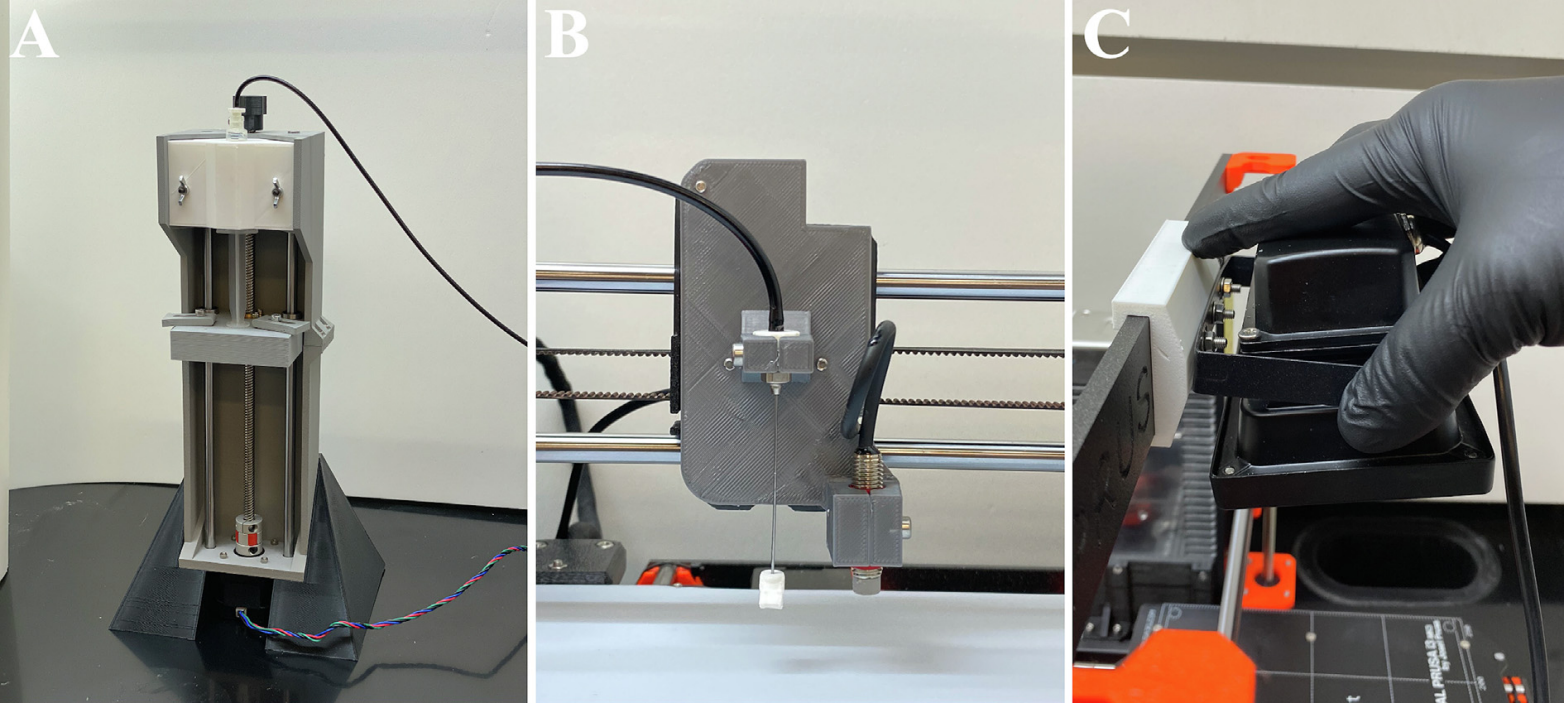
Installation of syringe, needle, Bowden tube, and UV curing lamp. (A) Image of the assembled SPECs syringe pump with a 5 ml syringe and Bowden tube installed. (B) The needle with shield and Bowden tube installed on the extruder head. (C) Attachment of the UV lamp onto the top cross member of the Prusa i3 mk3.
2. Attach the other end of the UV resistant tubing to the desired needle size using a male Luer lock hose barb connector (Fig. 5B).
3. Install the needle tip shield onto the needle by sliding it onto the needle (Fig. 5B). NOTE: The needle tip shield may need to be modified or glued in place depending on the needle size.
4. Attach the UV curing lamp to the UV light mount with three M3x8mm bolts and three M3 nuts. The UV light mount can then be snapped onto the top cross member of the Prusa i3 mk3 (Fig. 5C).
5. Cut to size and attach the Teflon sheet to the build platform of the printer using the adhesive backing.

### 5.5 Software modification

This system uses free software for modeling of parts, slicing STL models, and communicating with the printer. Begin by downloading and installing the following software:

1. Prusa Slic3r (slicing software)
2. Pronterface (printer communication software if the use of an SD card is undesirable)

#### 5.5.1 Prusa Slic3r software modification

A Prusa Slicer configuration bundle file is included in the Mendeley repository which will automatically make all of the adjustments outlined below to print using a 5 ml syringe with a 22-gauge needle. Note that these configurations should be treated as a baseline and adjustment to parameters inside of Prusa Slicer will be required for individual testing environments, materials used, needle sizes, and syringe sizes. Performance optimization steps are given in Section 6. The configuration bundle will install presets in Prusa Slicer to be used with this conversion. These presets are:

1. Install the configuration bundle by opening Prusa Slicer and navigating to File/Import/Import Config Bundle.
2. Once the configuration bundle is installed, you can select the settings profiles from the right-side drop-down menus for Print Settings, Filament Settings, and Printer Settings that match the desired printing function. The “I3 MK3 FLUID DEPOSITION CONVERSION” preset should be chosen for Printer Settings. This preset adds G-code to allow for cold extrusion. For the Print and Filament Settings, the “COLD EXTRUDE” preset provides baseline settings for 3D objects. The presets “COLD EXTRUDE THIN FILM” and “COLD EXTRUDE MESH” provide settings for printing thin solid films and mesh films optimized for 2 layers of material, respectively.

#### 5.5.2 Pronterface software modification

1. Connect the Prusa i3 MK3 to the computer via USB.
2. Select the com port that the printer is connected to in the top left corner and set the baud rate to 240,000.
3. Click on connect and wait for the prompt to display “connected”
4. If the prompt shows an error or unrecognizable symbols, change the baud rate and attempt to connect again.
5. Once you are connected to the printer, click on “load file” and select your G-code file that Prusa Slic3r created.
6. When you are ready to print, click on the print button.
7. Do not close Pronterface while the printer is running.

### 5.6 Optional – Trifold UV shield

UV eye protection and UV shielding should be used to protect users from UV exposure. If the user does not have any existing shield, the Trifold UV Shield included in these instructions is a low-cost solution.

1. Lay the 24″ × 24″, 1/8″ Thick Laser Shielding panel on a flat surface.
2. Position the two 24″ × 12″, 1/8″ Thick Laser Shielding panels on opposite sides of the 24″ × 24″, 1/8″ Thick Laser Shielding panel.
3. Place three of the folding butt hinges at equal spacings at each seam between the 24″ × 24″ and 24″ × 12″ panels.
4. Mark the locations of the holes in the hinges and remove.
5. Drill holes in the panels at the marked locations using a 4 mm drill bit.
6. Attach the hinges to the panels using the M3 × 12 mm Hex Bolts and M3 Hex Nuts.
7. Fold the side panels in at a 45° angle and stand up in front of the printing system.

## 6 Operation instructions

WARNING: UV EYE PROTECTION AND UV SHIELDING SHOULD BE USED TO PROTECT USERS FROM UV EXPOSURE.

### 6.1 Material loading

1. Unscrew the 2 M3 wing nuts and remove the syringe and remove the female Luer lock hose barb connector, if attached.
2. Fill the syringe with the required volume of fluid. It is recommended that the syringe is slightly overfilled to account for the volume of the nylon tube and needle and priming waste. NOTE: The required volume for a particular object is displayed in Prusa Slicer.
3. Reattach the female Luer lock hose barb connector, place the loaded syringe into the syringe pump clamps, and gently secure with 2 M3 wing nuts. NOTE: If the wing nuts are over tightened it can deform the syringe and cause squeeze out of material or lock up the motion of the syringe.
4. Raise the plunger plate using the lead screw knob so that it contacts the syringe plunger and clamp the plunger using the plunger clamps.
5. Secure the syringe plunger using the plunger clamps (Fig. 5A).
6. If there are any air bubbles in the resin, let the syringe sit for several minutes until the bubbles are observed to rise towards the nozzle. Tapping the syringe with a finger can facilitate this process.
7. Slowly rotate the lead screw knob until the nylon tube and needle are filled with resin and all air is ejected from the tubing and syringe needle.
8. The system is now primed and ready to print.

### 6.2 Preparation of 3D object file and execution of print.

1. Begin by opening your desired design to be printed in Prusa Slicer.
2. Select the correct presets as outlined in Section 5.5.1 and make any desired changes to the slicer parameters such as infill, shell thickness, or top/bottom layer thickness. It is recommended to print all solid parts using either 100% infill or a high enough perimeter count that it completely fills the model.
3. It is recommended to print with a skirt in order to ensure that fluid is flowing cleanly and at the desired height prior to beginning to print the object.
4. Export the g-code file to a known location.
5. Perform a mesh bed leveling prior to printing by following the steps outlined in the Prusa i3 mk3 user manual and adjust the needle to the desired height off of the print bed.
6. Connect Pronterface to the printer and load the gcode file as outlined in Section 5.5.2.
7. Click print on Pronterface to initiate the print.
8. Turn on the UV lamp, if applicable.
9. Closely monitor the first layer of the print and use the live Z adjust function of the Prusa i3 mk3 to adjust the first layer height if needed.
10. Once the print is complete, remove part from print bed and perform post-processing as specified by the build material documentation.
11. Clean the syringe, tubing, and needle by removing from the system and flushing with isopropyl alcohol.

## 7 Validation and characterization

**Fig. 6:**
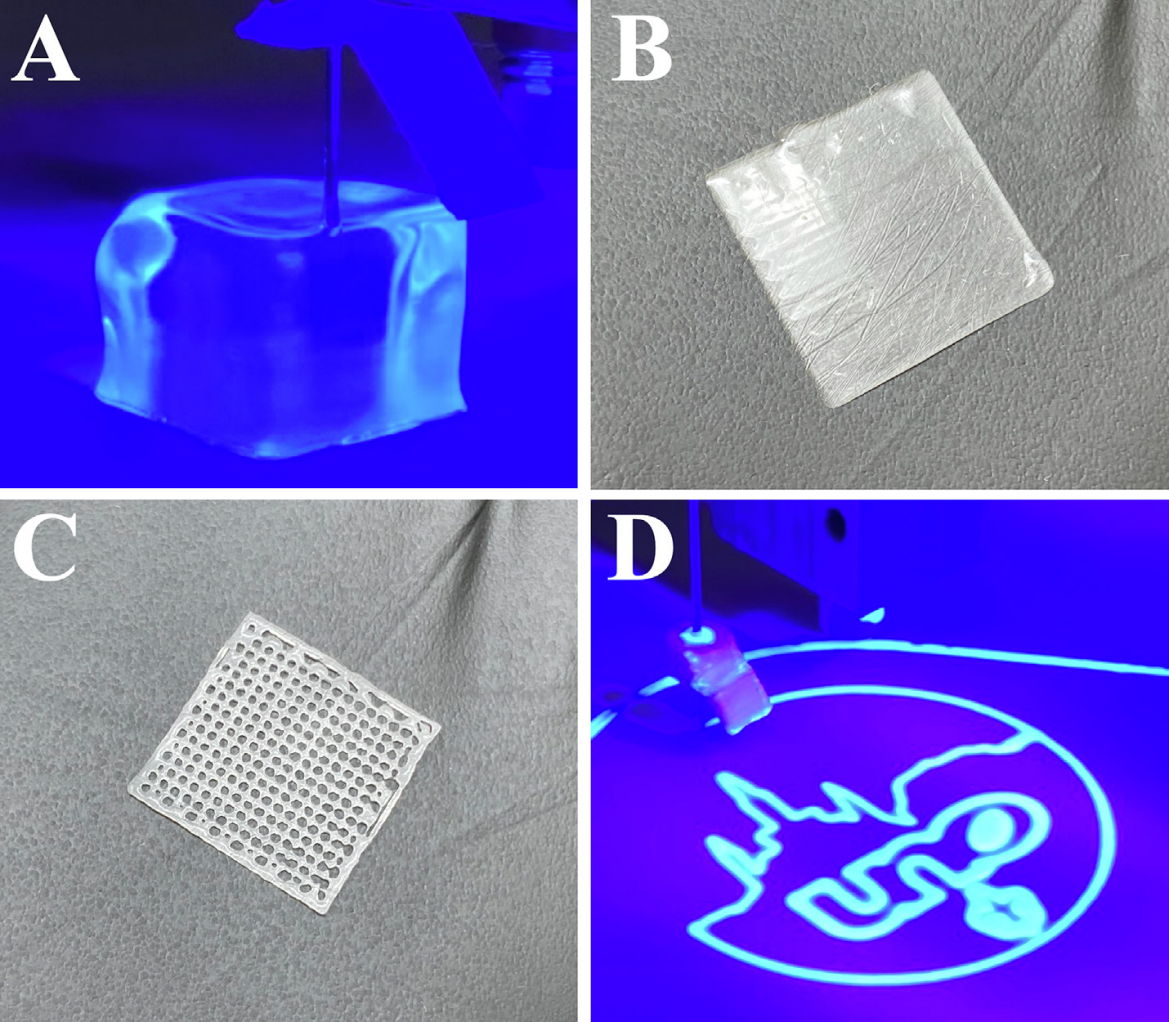
Images of printed objects. (A) Image of a 3D part being printed with a photopolymer resin and illuminate by the UV curing lamp. A completed 0.4 mm freestanding (B) solid and (C) mesh film printed using photopolymer resin. (D) Printing of the University of New Orleans logo.

Fig. 6 shows a series of objects produced to test the capabilities of the printer using UV curable photopolymer resins. Curing from the UV lamp was sufficient to produce 3D objects during the build process for clear SLA resin purchased from Formlabs and Anycubic. The dimensional accuracy of the printed objects is dependent on multiple factors including the material viscosity, needle size, syringe size, extrusion multiplier settings, and print speeds. Calibration and optimization were iteratively determined using Formlabs SLA resin, a 5 ml syringe, and a 22-gauge needle. A print speed, first layer height, and a layer height of 20 mm s^−1^, 0.1 mm, and 0.2 mm, respectively, were used for 3D objects (Fig. 6A). The retraction settings for length and Z-lift were both 1 mm and the length on restart was 0.001 mm. An extrusion multiplier setting of 0.075 was sufficient to allow adequate flow rates to deposit consistent layers. The retraction settings stated above are the slicer software settings for the use of filament and do not directly indicate the amount of material flow in the SPECS system as this will vary depending on syringe and needle size. The Prusa i3 mk3 with the SPECS modification was able to achieve an accuracy within ±0.1 mm of modeled dimensions along the X-, Y-, and Z-axis. The part accuracy was influenced by the spreading of material upon deposition prior to receiving sufficient UV exposure to impede fluid flow. Accuracy may be increased or reduced depending on the use of build material with greater or lower viscosity, respectively. For objects with a Z-height greater than approximately 4 mm, the geometric accuracy was reduced due to shadowing effects created by the syringe needle, extruder head, and syringe tip shield leading to uneven curing. The reduced accuracy depends on the specific geometry of the object; however, improvements can be made by adjusting the lamp position to a side position or by adding an additional lamp if taller objects are desired. With the UV lamp mounted on the upper cross member of the Prusa i3 mk3, partial curing of the Formlabs SLA resin occurred immediately upon deposition, which was sufficient for subsequent deposition of additional layers without the need to pause the print process. To reach full curing and optimization of mechanical properties, additional UV exposure of the printed objects was performed according to manufacturer recommendations; 30 min for Formlabs Clear SLA resin. While the SPECS system is limited to simple object geometries, more complex objects with overarching features could be possible by extending the SPECS design to include an additional syringe pump for a dissolvable support material.

This SPECS modified printing system is capable of producing 3D objects for a fraction of the cost of commercially available bioprinters, allowing for greater access of this manufacturing method. 3D printed parts for this conversion can be printed with the Prusa i3 mk3 prior to disassembly, eliminating the need for a second 3D printer for parts manufacturing. The conversion can be completed in hours, requires minimal hardware, and is non-destructive and reversable. With traditional SLA printers, a large amount of material must be used to fill the build tray. This system allows for a minimal material loss with only a thin film of uncured resin being lost in the cleaning process. The SPECS modification is beneficial for research and development with limited material quantities or high-cost materials.

## 8 Design files

Mendeley repository.

https://doi.org/10.17632/cv9cfj39b7.1.

## Declaration of Competing Interest

The authors declare that they have no known competing financial interests or personal relationships that could have appeared to influence the work reported in this paper.
